# The Luminescent Conjugated Oligothiophene h-FTAA Attenuates the Toxicity of Different Aβ Species

**DOI:** 10.1021/acs.biochem.1c00265

**Published:** 2021-09-01

**Authors:** Linnea Sandin, Simon Sjödin, Ann-Christin Brorsson, Katarina Kågedal, Livia Civitelli

**Affiliations:** † Experimental Pathology, Department of Clinical and Experimental Medicine, Faculty of Health Sciences, Linköping University, Linköping 581 83, Sweden; ‡ Division of Molecular Biotechnology, Department of Physics, Chemistry and Biology, Linköping University, Linköping 581 83, Sweden

## Abstract

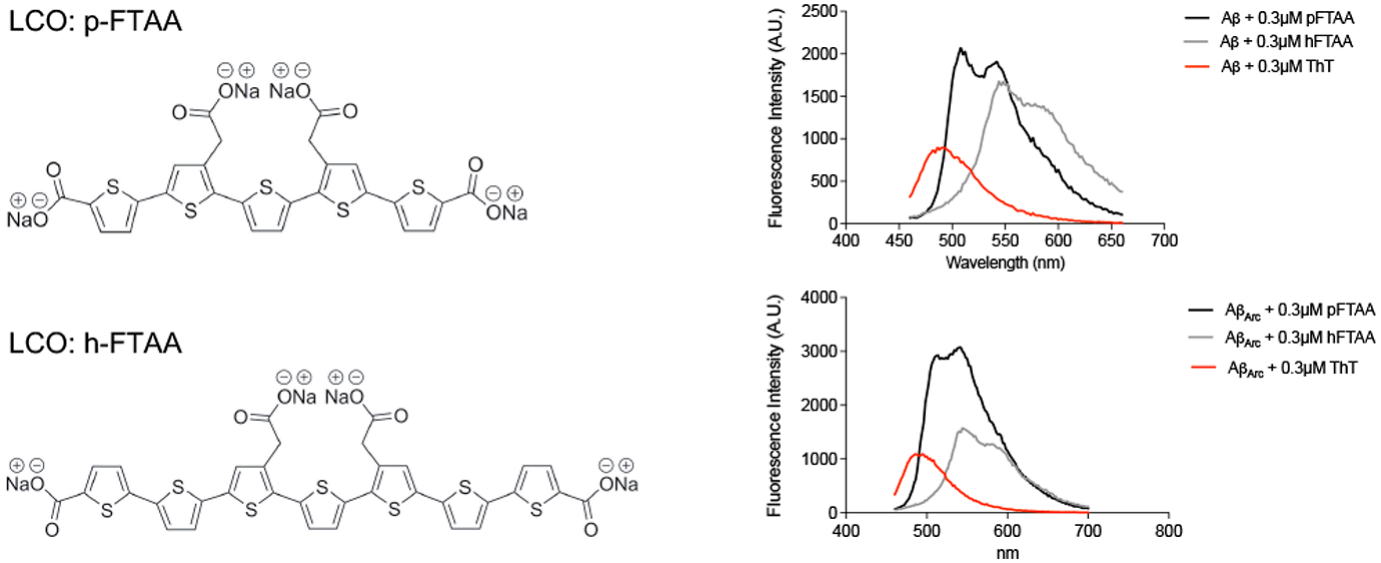

The prevailing opinion is that prefibrillar β-amyloid (Aβ) species, rather than end-stage amyloid fibrils, cause neuronal dysfunction in Alzheimer’s disease, although the mechanisms behind Aβ neurotoxicity remain to be elucidated. Luminescent conjugated oligothiophenes (LCOs) exhibit spectral properties upon binding to amyloid proteins and have previously been reported to change the toxicity of Aβ_1–42_ and prion protein. In a previous study, we showed that an LCO, pentamer formyl thiophene acetic acid (p-FTAA), changed the toxicity of Aβ_1–42_. Here we investigated whether an LCO, heptamer formyl thiophene acetic acid (h-FTAA), could change the toxicity of Aβ_1–42_ by comparing its behavior with that of p-FTAA. Moreover, we investigated the effects on toxicity when Aβ with the Arctic mutation (Aβ_Arc_) was aggregated with both LCOs. Cell viability assays on SH-SY5Y neuroblastoma cells demonstrated that h-FTAA has a stronger impact on Aβ_1–42_ toxicity than does p-FTAA. Interestingly, h-FTAA, but not p-FTAA, rescued the Aβ_Arc_-mediated toxicity. Aggregation kinetics and binding assay experiments with Aβ_1–42_ and Aβ_Arc_ when aggregated with both LCOs showed that h-FTAA and p-FTAA either interact with different species or affect the aggregation in different ways. In conclusion, h-FTAA protects against Aβ_1–42_ and Aβ_Arc_ toxicity, thus showing h-FTAA to be a useful tool for improving our understanding of the process of Aβ aggregation linked to cytotoxicity.

Alzheimer’s disease (AD) is a common progressive neurodegenerative disease associated with the extracellular accumulation of protein aggregates comprising fibrils of β-amyloid (Aβ). The formed extracellular senile plaques have long been considered the main toxic agent, but there is a growing consensus that smaller soluble Aβ aggregates such as oligomers exert neurotoxic activity because these correlate better with disease progression.^1,2^ The Aβ peptides are proteolytically cleaved from the amyloid precursor protein (APP) by β-secretase and γ-secretase into mainly Aβ_1–40_ or Aβ_1–42_. Aβ_1–42_ has a higher propensity to aggregate and is considered to be more toxic than the less hydrophobic Aβ_1–40_.^3^ There are several known mutations of APP and the presenilin proteins of the γ-secretase that give rise to AD, either via an increased level of production of Aβ or via its propensity to aggregate.^4−6^ One familial form of AD is the Arctic mutation of APP (E693G), which deviates from many other known APP mutations by the rapid formation of stable toxic Aβ oligomers. The pathogenesis associated with the Arctic mutation resembles that of wild type Aβ, but the progression is faster.^7−9^

Luminescent conjugated oligothiophenes (LCOs) are fluorescent probes of conjugated thiophenes with demonstrated amyloid binding capacity. An important property of the LCOs is the flexible backbone that possesses different optical properties when bound to different amyloid structures, which makes it possible to distinguish aggregates of diverse structure and content.^10^ For instance, the LCOs p-FTAA (pentamer formyl thiophene acetic acid) and h-FTAA, with two additional thiophene units (heptamer formyl thiophene acetic acid) (Figure 1), discriminate between Aβ and Tau, the hallmarks of AD.^11^ Moreover, spectral analysis has revealed that the LCOs also have the ability to identify prefibrillar species in comparison to commonly used dye molecules such as Thioflavin-T (ThT) and Congo red, which detect later end-stage amyloid fibrils.^11,12^ Because LCOs can interact with early soluble intermediates in the fibrillation pathway, it is conceivable that it might have an impact on Aβ aggregation. We recently demonstrated that p-FTAA can interact with Aβ_1–42_ and manipulate the aggregation process, leading to reduced cell toxicity.^13^

**Figure 1:**
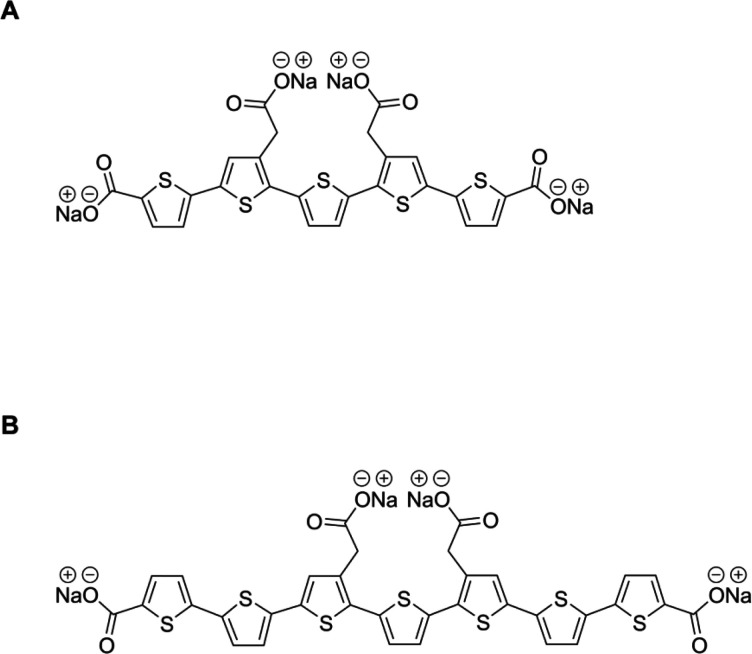
Chemical structures of the anionic luminescent conjugated oligothiophenes p-FTAA and h-FTAA. (A) Pentamer formyl thiophene acetic acid (p-FTAA) contains five conjugated thiophene rings and four carboxyl groups. (B) Heptamer formyl thiophene acetic acid (h-FTAA) contains seven conjugated thiophene rings and four carboxyl groups.

Here we aimed to investigate whether the LCO h-FTAA could be used to manipulate toxicity induced by Aβ_1–42_ and, in addition, by Aβ_Arc_. We discovered that h-FTAA could rescue Aβ_1–42_-mediated toxicity more effectively than p-FTAA and that h-FTAA also conferred protection when aggregated with Aβ_Arc_, which most probably is due to stronger binding of Aβ_Arc_ to h-FTAA than to p-FTAA. This is evident from spectral binding studies that revealed a greater capacity of h-FTAA to bind to Aβ_Arc_ compared to that of p-FTAA. In light of our previous study, our data highlight that h-FTAA is a more versatile molecule than p-FTAA and shows some potential to deepen the mechanistic behavior of Aβ species aggregation.

## Materials and Methods

### Peptide Preparation

Recombinant Aβ_1–42_ (rPeptide) was dissolved in trifluoroacetic acid, which was removed by lyophilization. The peptide was then dissolved once more in 1,1,1,3,3,3-hexafluoro-2-propanol (HFIP), aliquoted, and then lyophilized. The Aβ_1–42_ E22G peptide (Arctic peptide, Bachem) was dissolved in trifluoroacetic acid (TFA), sonicated on ice for 30 s, and subsequently frozen in liquid nitrogen before the TFA was removed by lyophilization. The peptide was redissolved in HFIP, aliquoted, frozen in liquid nitrogen, and lyophilized. Both peptides were kept at −80 °C. Prior to each experiment, aliquots of Aβ_Arc_ and Aβ_1–42_ were dissolved in 2 mM NaOH to a final concentration of 222 μM.

### Luminescent Conjugated Oligothiophenes (LCOs)

The LCOs h-FTAA and p-FTAA, a generous gift from Prof. Peter Nilsson, were synthesized as described previously.^11,14^ Briefly, after the reaction had reached completion, both h-FTAA and p-FTAA precipitates were collected by centrifugation, lyophilized, redissolved in water at a concentration of 1.5 mM, and stored under refrigerated, dark conditions without further purification.

### Cell Culturing and Viability Measurements

SH-SY5Y neuroblastoma cells (ECACC, Sigma-Aldrich) were cultured in minimal essential medium (MEM) GlutaMAX (Life Technologies) supplemented with 10% fetal calf serum (FCS, PAA Laboratories), 2 mM l-glutamine, 50 units/mL penicillin, and 50 μg/mL streptomycin (all from Lonza) at 37 °C in 5% CO_2_. Prior to experiments, the cells were differentiated for 7 days in serum medium by 10 μM retinoic acid (Sigma-Aldrich). The cells were trypsinized, seeded in a 96-well culture plate at a density of 30000 cells/well, and subsequently exposed to both Aβ_1–42_ and Aβ_Arc_. Before exposure, Aβ_Arc_ was diluted to a concentration of 10 μM with or without 10, 1, or 0.1 μM p-FTAA or h-FTAA and allowed to aggregate for 0, 1, 2, and 4 h at 37 °C. At the respective times, Aβ_Arc_ samples were further diluted in serum free medium to a concentration of 3 μM and exposed to neuroblastoma cells for 72 h. The same procedure was applied to Aβ_1–42_ that was diluted to a concentration of 10 μM with or without 3 μM p-FTAA or h-FTAA and allowed to aggregate for ≤5 h at 37 °C. At the respective times, samples were further diluted in serum free medium to a concentration of 3 μM and exposed to neuroblastoma cells for 72 h. Cells were morphologically examined in a phase contrast microscope, and viability was determined using the XTT assay (Roche Diagnostics) according to the manufacturer’s instructions.

### Aβ Aggregation Kinetics

Emission was recorded with Aβ_1–42_ and Aβ_Arc_ diluted to concentrations of 10 and 0.3 μM of p-FTAA and h-FTAA before their addition to a 96-well microtiter plate (Corning Inc. Life Sciences) in 10 mM phosphate buffer with 14 mM NaCl and 2.7 mM KCl (PBS pH 7.4). The *in situ* change in fluorescence from the probes in the presence of Aβ_1–42_ and Aβ_Arc_ was verified using a Tecan Safire2 multiplate reader recording an emission spectrum between 460 and 700 nm, at an excitation wavelength of 440 nm in a quiescent state at 37 °C.

### Binding Assay

Excitation was recorded to study the ratio of bound and free p-FTAA or h-FTAA over time. Aβ_1–42_ and Aβ_Arc_ were used at concentrations of 10 and 0.3 μM of p-FTAA or h-FTAA and added to a 96-well microtiter plate (Corning Inc. Life Sciences). The fluorescence at 515 nm for p-FTAA and 545 nm for h-FTAA was recorded using an excitation spectrum from 380 to 500 nm.

### Transmission Electron Microscopy

Aβ_1–42_ and Aβ_Arc_ were incubated at 37 °C for 3 and 0 h, respectively, at 10 μM without or with 3 or 10 μM LCOs. The samples were collected after gently mixing the aggregated solution and then added to Formvar/carbon-coated 400 mesh copper transmission electron microscope grids (Agar Scientific) by placing 10 μL of the sample fluid on the grid for 2 min and subsequently wiping it off. Staining was performed using 10 μL of 4% uranyl acetate for 2 min, and then the grid was blotted dry. The grids were analyzed with a JEOL JEM1230 transmission electron microscope (Akishima) equipped with a SC1000 ORIUS CCD camera and DigitalMicrograph (DM) version 1.71.38 (Gatan).

### Statistics

Statistics were calculated using one-way analysis of variance (ANOVA) with a Bonferroni post hoc test. Statistics for the aggregation kinetics were calculated using two-way ANOVA with Geisser–Greenhouse correction. Statistical analyses were performed using GraphPad Prism 5 (GraphPad Software Inc.). Differences were considered significant for *p* values of <0.05 (one asterisk), <0.01 (two asterisks), and <0.001 (three asterisks). Bar graphs represent means ± the standard deviation.

## Results and Discussion

### h-FTAA but Not p-FTAA Protects against Toxicity from Aβ_1–42_ and Aβ_Arc_

**Figure 2:**
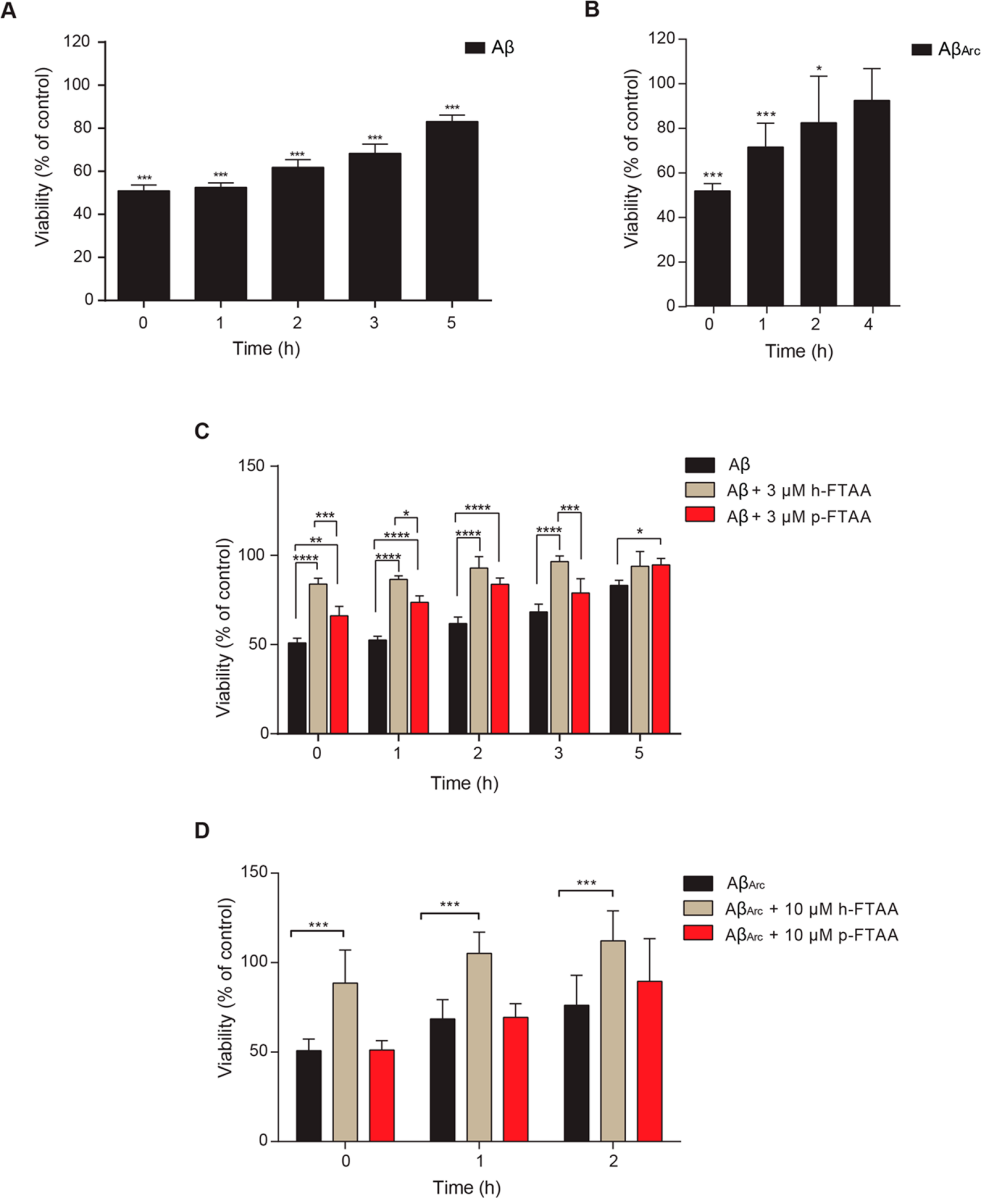
h-FTAA attenuated both Aβ_1–42_ and Aβ_Arc_ toxicity. (A) Aβ_1–42_ (10 μM) was aggregated for 0, 1, 2, 3, and 5 h at 37 °C, diluted to 3 μM, and exposed to SH-SY5Y neuroblastoma cells for 72 h. Cell viability was assessed using the XTT assay (*n* = 12). (B) Aβ_Arc_ (10 μM) was aggregated for 0, 1, 2, or 4 h at 37 °C, diluted to 3 μM, and exposed to SH-SY5Y neuroblastoma cells for 72 h. Cell viability was assessed using the XTT assay (*n* = 12). (C) Aβ_1–42_ (10 μM) was aggregated with or without 3 μM h-FTAA or p-FTAA for 0, 1, 2, 3, and 5 h at 37 °C, diluted to 3 μM, and exposed to neuroblastoma cells for 72 h. Cell viability was assessed using the XTT assay (*n* = 8). (D) Aβ_Arc_ (10 μM) was aggregated with or without 10 μM h-FTAA or p-FTAA for 0, 1, or 2 h at 37 °C, diluted to 3 μM, and exposed to neuroblastoma cells for 72 h. Cell viability was assessed using the XTT assay (*n* = 8).

The generation of Aβ oligomers has emerged to be crucially involved in the onset and progression of AD.^2^ We recently demonstrated that the LCO p-FTAA reduces the toxicity of Aβ_1–42_ by generating larger species that are resistant to degradation and less prone to propagate.^13^ The Arctic APP (Aβ_Arc_) mutation displays a typical clinical picture of AD but with a substantially more rapid formation of soluble oligomers compared to that for wild type APP.^7−9^ To investigate whether the binding of the LCOs h-FTAA and p-FTAA could have an impact on the toxicity of different forms of Aβ (Aβ_1–42_ and Aβ_Arc_) during the process of aggregation, the cell viability XTT assay was used. Human SH-SY5Y neuroblastoma cells were exposed to Aβ_1–42_ species generated at different times during the aggregation process. Exposed cells had reduced cell viability at 0 h of ∼50%, but Aβ was still toxic at all of the time points investigated (Figure 2A). Cells exposed to Aβ_Arc_ immediately after dissolving the peptide (aggregation for 0 h) decreased the cellular viability to ∼50% of that of the control (Figure 2B). The toxic effect persisted after aggregation at 1 and 2 h but was completely abolished after aggregation for 4 h. This result demonstrates that the components necessary for neuroblastoma cell toxicity exist early in the aggregation process of Aβ_Arc_ and decrease with time, indicating that early formed species rather than fibrils exert the detrimental effects. The same behavior is shown by the Aβ peptide, but the pool of toxic Aβ species shows an elongated cytotoxic effect. Whalen et al. showed that Aβ_Arc_ induces toxic effects in mouse neuronal cells 24 h after exposure, while Aβ gives the same effect after 96 h, which shows the fast and slow toxicity behavior of the two different Aβ isoforms.^15^

Manipulating the levels of soluble prefibrillar species and the aggregation pathway might be a valid approach for modifying the toxicity of the amyloid peptides and their aggregates. The LCOs have previously been shown to interact with and detect prefibrillar species of several amyloid proteins, including the Aβ peptide (Aβ_1–42_),^11,12,14^ and to stabilize prion protein aggregates, which prevented infectiousness of prion protein.^16^ For this purpose, the study of the behavior of another LCO, such as h-FTAA, is important to expand the range of molecules that could potentially provide us with useful information about the Aβ pathway of aggregation. Therefore, Aβ_1–42_ was aggregated in the presence of both h-FTAA and p-FTAA, using a concentration of 3 μM as previously reported.^13^ In this case, h-FTAA could rescue Aβ_1–42_ toxicity for cell exposures of ≤3 h and p-FTAA could also rescue Aβ_1–42_ toxicity but to a lesser extent compared to h-FTAA (Figure 2C). Our recent publication demonstrated that the LCO p-FTAA is very effective against Aβ_1–42_ toxicity, but here, we discovered that h-FTAA is a quite powerful molecule that can reduce the toxicity of Aβ_1–42_ significantly with respect to p-FTAA for ≤3 h.

When cells were exposed to Aβ_Arc_ aggregated at 10 μM, with or without 10 μM h-FTAA for 0, 1, and 2 h, a protective effect was accomplished at all times investigated (Figure 2D). No protective effect using lower concentrations of h-FTAA (1 and 0.1 μM) was noticed after any period of exposure (data not shown). The aggregation of Aβ_Arc_ was conducted in the presence of p-FTAA (10 μM) using the same conditions as stated above for h-FTAA, but p-FTAA was unable to confer protection to the cells as shown in Figure 2D. These data demonstrate that at an equimolar concentration of Aβ_Arc_ and h-FTAA, the cytotoxic effect is completely abolished. Hence, h-FTAA might sequester toxic species present early in the aggregation process and/or promote the formation of ordered and less toxic Aβ assemblies as previously demonstrated with Aβ_1–42_ and p-FTAA.^13^ Interestingly, co-aggregation of p-FTAA with Aβ_Arc_ did not rescue the cells, indicating that the protective effect of h-FTAA could be due to the extension of the thiophene backbone, with two additional thiophene rings within the h-FTAA molecule.

**Figure 3:**
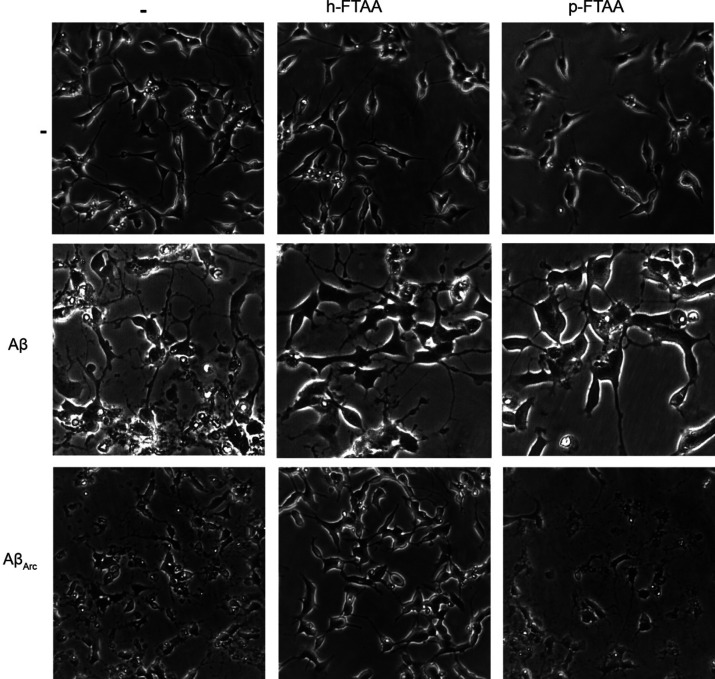
h-FTAA rescues both Aβ_1–42_ and Aβ_Arc_ toxicity in neuroblastoma cells. Phase contrast images of cells exposed to 3 μM Aβ_1–42_ or Aβ_Arc,_ at 0 h, with or without h-FTAA (10 μM) or p-FTAA (3 μM). Scale bar of 50 μm.

**Figure 4:**
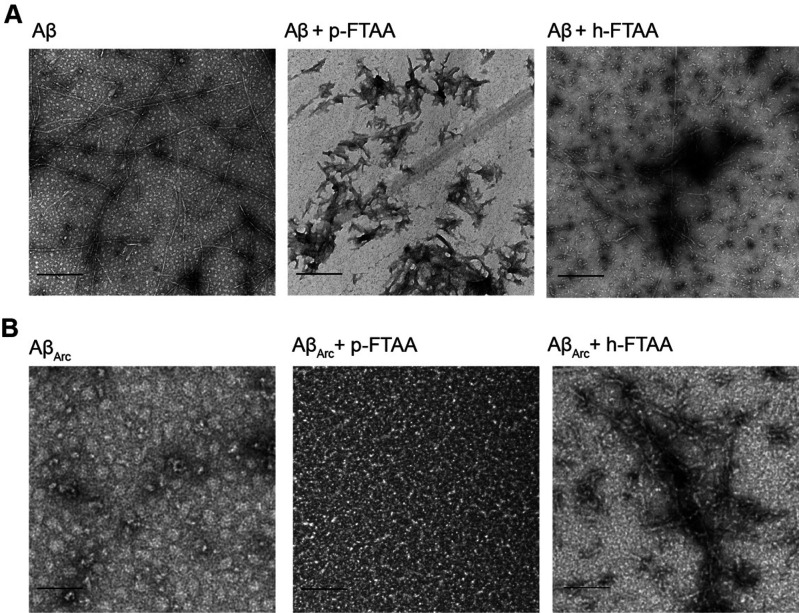
Fibril morphology of Aβ_1–42_ and Aβ_Arc_ aggregated without or with LCOs. (A) TEM images of Aβ_1–42_ aggregated without or with p-FTAA and h-FTAA at 3 h. (B) TEM analysis of Aβ_Arc_ aggregated without or with p-FTAA and h-FTAA at 0 h. Scale bars of 500 nm.

As cell morphology analysis with phase contrast microscopy makes evident, neuroblastoma cells that were exposed to Aβ co-aggregated with either h-FTAA or p-FTAA showed the complete absence of the typical morphological changes for neurotoxicity, such as the breakdown of cell processes and the appearance of shrunken cell bodies as an indication of significant cell loss. When cells were exposed to Aβ_Arc_, they appeared to be shrunken with retracted dendrites both without and with p-FTAA, whereas cell exposure to Aβ_Arc_ aggregated in the presence of h-FTAA completely rescued the phenotype (Figure 3). These data confirm the result obtained on cell toxicity as shown in Figure 2 and demonstrate the specific ability of h-FTAA to rescue Aβ_1–42_- and Aβ_Arc_-induced cytotoxicity. To further study what can drive the ability of the LCOs to rescue the toxicity induced by Aβ_1–42_ and Aβ_Arc_, we conducted transmission electron microscopy (TEM) analysis for Aβ_1–42_ aggregated without and with LCOs at the key time point of 3 h. First, we noticed that Aβ_1–42_ showed the formation of fibrils, but with a component of oligomers still present in the sample. However, Aβ_1–42_ aggregated with the LCOs did not show the presence of oligomers, but Aβ_1–42_ aggregated with p-FTAA gave rise to fibrils that had an amorphous structure. On the contrary, Aβ_1–42_ aggregated with h-FTAA showed the formation of extended fibrils along with the presence of very short and less branched filaments. Overall, TEM images of Aβ_1–42_ aggregated with the LCOs showed that fibrils formed in all of the samples, but with differences in the three-dimensional structure (Figure 4A). When we analyzed Aβ_Arc_ aggregated without and with LCOs at the key time point of 0 h, we could notice that any aggregated structures were formed with Aβ_Arc_ alone or Aβ_Arc_ aggregated with p-FTAA, while Aβ_Arc_ aggregated with h-FTAA showed the presence of protofibrils. Then, through TEM images, we could reveal that h-FTAA induces a rapid change in Aβ_Arc_ aggregation toward fibril formation, and this result might explain the rescue of the cytotoxic ability of h-FTAA when it is used in combination with Aβ_Arc_. Preliminary data on the levels of soluble and insoluble Aβ_1–42_ and Aβ_Arc_ species collected at different time points during the aggregation process reveal that both p-FTAA and h-FTAA can accelerate the process of fibrillation (data not shown).

Two plausible mechanisms for how hydrophobic molecules could interfere with aggregation have been proposed; by stabilizing monomers and preventing β-structure formation and thereby preventing oligomerization or by increasing the level of formation of β-structure and accelerating the nucleation rate and hence reducing the time of exposure to toxic aggregation intermediates.^17^ The latter proved to be the mechanism behind the reduced toxicity observed with p-FTAA and Aβ_1–42_.^13^ It is conceivable that the mechanisms that protect h-FTAA from Aβ_Arc_-induced toxicity are associated with the mechanisms discovered with p-FTAA for Aβ_1–42_.

### Aggregation Kinetics of Aβ_1–42_ and Aβ_Arc_

**Figure 5:**
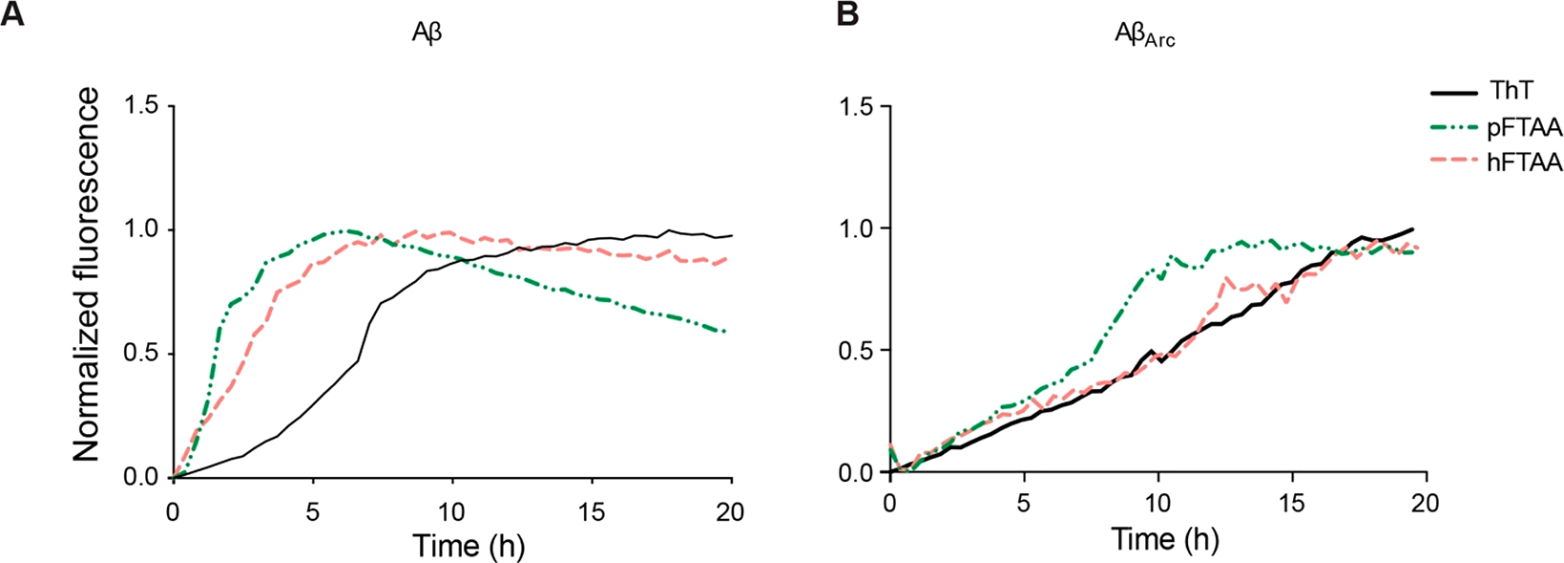
Different morphologies and kinetics of Aβ_1–42_ and Aβ_Arc_ with and without p-FTAA and h-FTAA. (A) Aβ_1–42_ (10 μM) or (B) Aβ_Arc_ (10 μM) was aggregated with 0.3 μM p-FTAA, h-FTAA, and ThT in 10 mM phosphate buffer with 14 mM NaCl and 2.7 mM KCl (PBS pH 7.4) at 37 °C in a quiescent state over time. The emission at 480 nm for ThT, 510 nm for p-FTAA, and 550 nm for h-FTAA at an excitation wavelength of 440 nm is shown (*n* = 3).

To elucidate the mechanism behind the diverging results in protection against Aβ toxicity between h-FTAA and p-FTAA, the aggregation of Aβ_1–42_ (10 μM) was monitored in the presence of the LCOs and ThT (0.3 μM), which is known to detect fibrillar Aβ species and frequently used to study the aggregation of proteins *in vitro*.^18^ As previously reported,^13^ the p-FTAA signal did not show any lag phase, but it rapidly started with the elongation phase with a peak around 5 h (Figure 5A). The LCO h-FTAA also did not show a lag phase, but the elongation phase arose more gently with respect to p-FTAA. On the contrary, the ThT fluorescence signal had a 6 h lag phase, followed by an increase in the signal that peaked after aggregation for 10 h and then reached a steady state plateau that remained constant up to 20 h. Both LCOs showed an absence in the lag phase if compared to ThT, but the elongation phase turned out to be steeper for p-FTAA than for h-FTAA. This difference in the kinetic behavior between the two LCOs could explain the better rescuing h-FTAA ability as shown by the cell toxicity data, highlighting the possibility that LCOs bind to toxic Aβ species that are formed at early time points during the aggregation in a different manner. The kinetic curves showing the single replicates for Aβ_1–42_ with the LCOs demonstrate that the aggregation behavior is reproducible and lead to statistical differences when Aβ_1–42_ is aggregated with p-FTAA with respect to h-FTAA (Figure S1, *p* < 0.0001). Table 1 shows the kinetic parameters, which confirm that samples of Aβ_1–42_ aggregated with both LCOs have a faster lag phase with respect to ThT and the aggregation kinetics reach the half-time (*t*_0.5_) more rapidly, meaning that the LCOs are very efficient in inducing the polymerization events. In conclusion, both p-FTAA and h-FTAA exhibited a logarithmic phase that occurred at different times, but whether this indicates that p-FTAA and h-FTAA bind to the same species, but these species appear earlier with p-FTAA, or that the LCOs bind to different species that appear during the aggregation process is inconclusive.

**Table 1:** Kinetic Parameters for the Aggregation of Aβ_1-42_ with ThT and/or the LCOs

	Aβ_1–42_ with ThT	Aβ_1–42_ with p-FTAA	Aβ_1–42_ with h-FTAA
lag time (h)	3.99	0.95	1.39
*t*_0.5_ (h)	6.35	1.31	2.47
*k*	0.29	1.87	0.63

When the aggregation of Aβ_Arc_ was monitored with fluorescence from both LCOs and ThT, a similar change in fluorescence over time was detected for h-FTAA and ThT during the first 11 h of aggregation where the signal increased linearly (Figure 5B). The signal from p-FTAA also showed a linear increase during the first 7 h but then displayed a tendency to a logarithmic phase between 7 and 10 h that reached a plateau after 11 h. This logarithmic phase also occurred for h-FTAA between 11 and 13 h but not for ThT. During the first period of aggregation, where Aβ_Arc_ is toxic for at least up to 2 h, the fluorescence curves of p-FTAA and h-FTAA showed almost identical behavior. Hence, the toxicity results cannot be explained by aggregation kinetics. Nevertheless, the experiment was performed for 20 h, and after 5 h, the curves start to differentiate, which indicates that either p-FTAA and h-FTAA bind to different species or the LCOs affect aggregation differently. Replicates for the aggregation kinetics of Aβ_Arc_ with the LCOs are statistically significant, thus confirming the differences in behavior for p-FTAA and h-FTAA (Figure S1, *p* < 0.0001). Table 2 shows the kinetic parameters for Aβ_Arc_ aggregated with p-FTAA. However, it was not possible to extrapolate the same parameters for Aβ_Arc_ aggregated with h-FTAA and/or ThT, because the curves became non-sigmoidal in the time frame of the performed experiment. The tendency toward a linear behavior in the aggregation process detected by h-FTAA, p-FTAA, and ThT most probably indicates that Aβ_Arc_ does not follow the typical nucleation seeding theory.^19^ Hence, fibrillization might not depend on nucleation seeds, or alternatively, seeds are present from the start of the experiment.

**Table 2:** Kinetic Parameters for the Aggregation of Aβ_Arc_ with ThT and/or the LCOs

	Aβ_Arc_ with ThT	Aβ_Arc_ with p-FTAA	Aβ_Arc_ with h-FTAA
lag time (h)	n/a	3.71	n/a
*t*_0.5_ (h)	n/a	7.30	n/a
*k*	n/a	0.1914	n/a

### h-FTAA and p-FTAA Bind Aβ_Arc_ Differently

**Figure 6:**
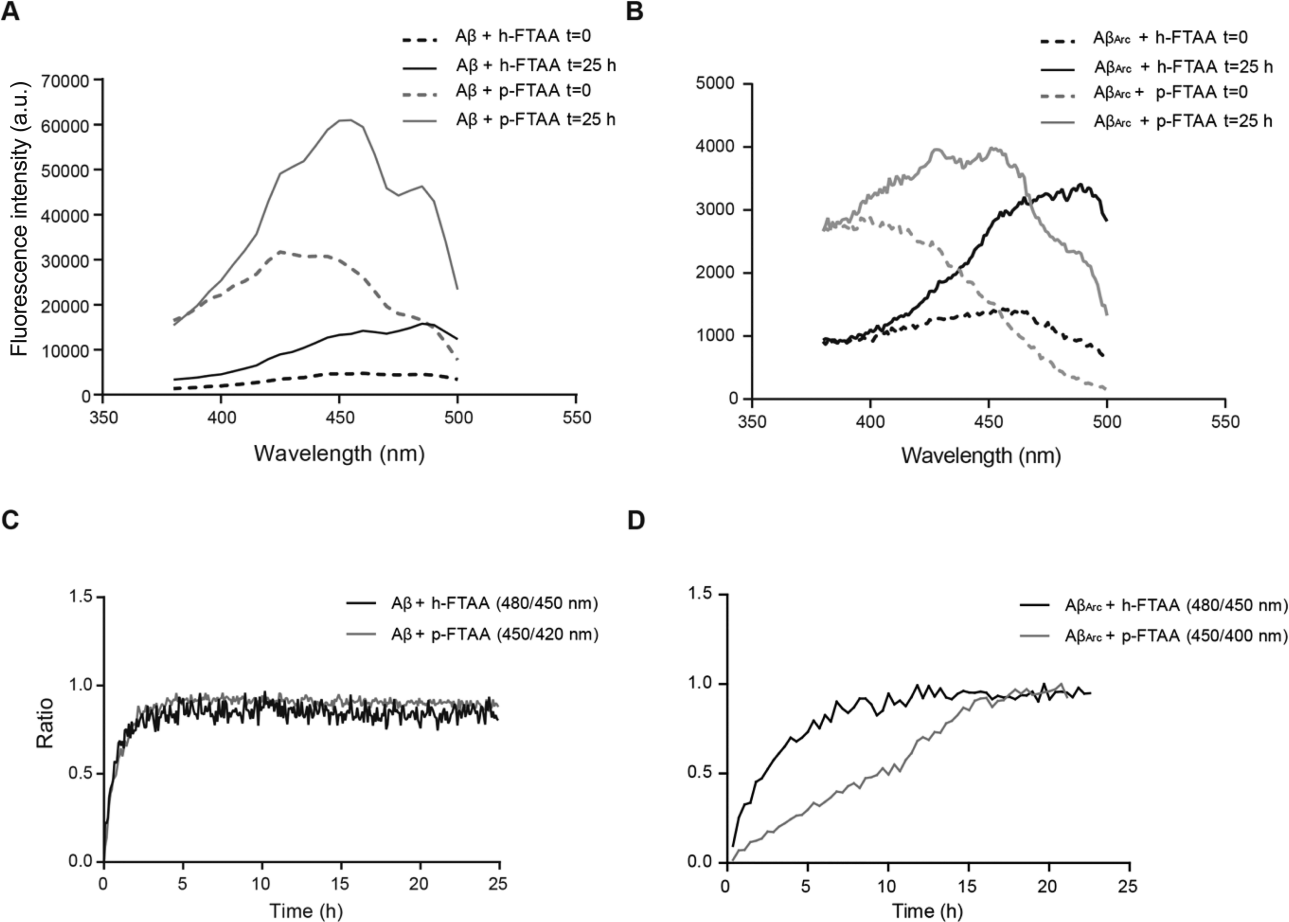
Binding of h-FTAA and p-FTAA to Aβ_1–42_ and Aβ_Arc_. (A) Aβ_1–42_ (10 μM) was probed with 0.3 μM h-FTAA or p-FTAA as described above. The fluorescence at the start and the end of the aggregation is shown. The emission at 545 nm for h-FTAA was recorded using an excitation spectrum from 380 to 500 nm. The emission at 515 nm for p-FTAA was recorded using an excitation spectrum from 380 to 500 nm. (B) Aβ_Arc_ (10 μM) was probed with 0.3 μM h-FTAA or p-FTAA at 37 °C in a quiescent state over time. The fluorescence at the start and the end of the aggregation is shown. (C) Excitation ratios of h-FTAA and p-FTAA when bound to Aβ_1–42_ (at 480 and 450 nm for h-FTAA and p-FTAA, respectively) against free h-FTAA at 450 nm or free p-FTAA at 420 nm, measured for emission at 545 and 515 nm for h-FTAA and p-FTAA, respectively, plotted over time (*n* = 3). (D) Excitation ratios of h-FTAA and p-FTAA when bound to Aβ_Arc_ (at 480 and 450 nm for h-FTAA and p-FTAA, respectively) against free h-FTAA at 450 or 400 nm for free p-FTAA, measured for emission at 545 and 515 nm for h-FTAA and p-FTAA, respectively, plotted over time (*n* = 3).

The decreased toxic effect of both Aβ_1–42_ and Aβ_Arc_ when aggregated together with h-FTAA but not p-FTAA prompted us to investigate the binding of h-FTAA and p-FTAA to the two different forms of Aβ. This was achieved by measuring the shift in the excitation wavelength that occurs when h-FTAA and p-FTAA bind to both Aβ_1–42_ and Aβ_Arc_. The excitation spectra of p-FTAA and h-FTAA were recorded at emission wavelengths of 515 and 545 nm, respectively, when binding to Aβ_1–42_. As shown in Figure 6A, for h-FTAA, the highest fluorescence intensity at the start of the aggregation occurred at 450 nm, representing free h-FTAA, while at the end of the aggregation, it was shifted to 480 nm, representing bound h-FTAA. For p-FTAA, the highest fluorescence intensity at the start of the aggregation occurred at 400 nm, representing free p-FTAA. With time, the intensity was shifted, and at the end of the aggregation, the highest intensity was found at 450 nm, representing p-FTAA bound to Aβ_Arc_ (Figure 6A). Interestingly, the graph with the plotted ratio of bound and free h-FTAA and p-FTAA over time showed an almost identical behavior for the two LCOs with a rapid increase that reached a plateau around 5 h (Figure 6C). As shown in Figure 6B, the behavior of both h-FTAA and p-FTAA was like that obtained when the LCOs were bound to Aβ_Arc_. When the ratio of bound and free h-FTAA is plotted over time, the data revealed an immediate initiation of a logarithmic phase that reached a plateau after 10 h, indicating no further binding (Figure 6D). The curve representing the ratio of bound and free p-FTAA over time showed a slower and more linear increase compared to the h-FTAA curve. The rapid increase in the ratio for h-FTAA indicates a higher binding affinity of h-FTAA for the formed aggregates compared to that of p-FTAA (Figure 6D). Notably, the binding affinity for aggregates that are formed within the first 2 h, where a toxic effect that could be rescued by h-FTAA was detected, is higher for h-FTAA than for p-FTAA. This observed differences in binding affinity could explain why h-FTAA conferred protection against toxicity when aggregated with Aβ_Arc_, whereas p-FTAA did not. The same experiment was performed by recording the excitation spectrum of LCOs while binding to Aβ_1–42_. This indicates that both p-FTAA and h-FTAA can bind early formed aggregates of Aβ_1–42_ with a binding affinity needed to prevent toxicity, confirming the data obtained with cell toxicity where a rescue effect was detected for both LCOs.

In conclusion, our findings demonstrate that h-FTAA, but not p-FTAA, could rescue the toxic effect of both Aβ_1–42_ and Aβ_Arc_, which could be explained by the affinity of h-FTAA for toxic prefibrillar species being better than that of p-FTAA. Molecules that bind and modify the characteristics of prefibrillar species have great potential to provide insight into the mechanism of toxicity and Aβ aggregation.

## Supporting Information Available

Graph of triplicate traces of the aggregation kinetics of Aβ_1–42_ and Aβ_Arc_ with p-FTAA and h-FTAA (Figure S1) (PDF)
