# Supplementary material for: The Luminescent Conjugated Oligothiophene h-FTAA Attenuates the Toxicity of Different Aβ Species
Source: Biochemistry. 2021 Sep 1;60(37):2773–80. doi: 10.1021/acs.biochem.1c00265 (PMC8459454; doi:10.1021/acs.biochem.1c00265)
Supplement: Supplementary file 1 — bi1c00265_si_001.pdf [file bi1c00265_si_001.pdf]

Supplementary information for: The luminescent conjugated oligothiophene h-FTAA attenuates toxicity of different Aβ species

Linnea Sandin[a], Simon Sjödin[a], Ann-Christin Brorsson[b], Katarina Kågedal[a], Livia Civitelli[a*]

[a]Experimental Pathology, Department of Clinical and Experimental Medicine, Faculty of Health Sciences, Linköping University, Linköping, 581 83 Sweden.
[b]Division of Molecular Biotechnology, Department of Physics, Chemistry and Biology, Linköping University, Linköping, 581 83 Sweden.

*To whom correspondence should be addressed:

Livia Civitelli, Nuffield Department of Clinical Neurosciences, John Radcliffe Hospital, West Wing, University of Oxford, Oxford, OX3 9DU United Kingdom, livia.civitelli@ndcn.ox.ac.uk

Supplemental Figure 1

Total number of pages: 2

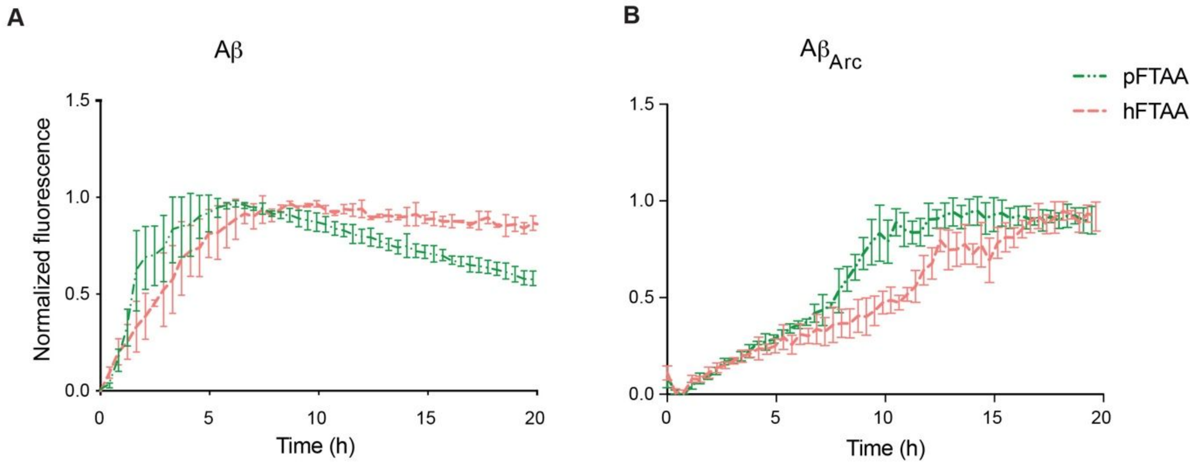

Figure S1. Aggregation kinetics of $A\beta_{1-42}$ and $A\beta_{Arc}$ with p-FTAA and h-FTAA. The graphs show the aggregation kinetic experiment of $A\beta_{1-42}$ (A) and $A\beta_{Arc}$ (B) peptides with p-FTAA and h-FTAA where error bars correspond to standard deviations calculated for the triplicates, ($p<0.0001$ $A\beta_{1-42}$ p-FTAA vs h-FTAA; $p<0.0001$ $A\beta_{Arc}$ p-FTAA vs h-FTAA).
